# Factors Associated With Alcohol Use After Metabolic and Bariatric Surgery: Protocol for an Ecological Momentary Assessment

**DOI:** 10.2196/87209

**Published:** 2026-01-14

**Authors:** Lisa R Miller-Matero, Daniel Saleh, Brittany Christopher, Maha Albujuq, Alyssa Vanderziel, Erin N Haley, Jordan M Braciszewski, Roland S Moore, Arthur M Carlin, Kristina M Jackson

**Affiliations:** 1 Behavioral Health Henry Ford Health System Detroit, MI United States; 2 Center for Health Policy and Health Services Research Henry Ford Health System Detroit, MI United States; 3 Michigan State University East Lansing, MI United States; 4 Pacific Institute For Research and Evaluation Berkeley, CA United States; 5 Department of Surgery Henry Ford Health System Detroit, MI United States; 6 Department of Psychiatry Robert Wood Johnson Medical School Rutgers, The State University of New Jersey New Brunswick, NJ United States

**Keywords:** metabolic and bariatric surgery, alcohol use, affect, disordered eating, ecological momentary assessment

## Abstract

**Background:**

Individuals who undergo metabolic and bariatric surgery (MBS) are at increased risk for postoperative alcohol use disorder. Reducing postoperative alcohol use could prevent the development of alcohol use disorder; however, the factors leading to episodic alcohol use are not known.

**Objective:**

The purpose of this paper is to describe the protocol for a study that will examine distal and proximal factors associated with episodic alcohol use and hazardous alcohol use among individuals who undergo MBS.

**Methods:**

We will enroll 100 participants who undergo MBS at a single health care system. Participants will complete measures of substance use, psychiatric symptoms, and disordered eating behaviors at baseline and at 6- and 12-week follow-ups. Participants will also complete a 3-week ecological momentary assessment protocol in which they will complete brief surveys each morning and evening, reporting on their mood, disordered eating, and substance use.

**Results:**

This study received funding from the National Institute on Alcohol Abuse and Alcoholism (R21 AA029423) in May 2023. This part of the grant was approved by the institutional review board in March 2024, and data collection occurred between November 2024 and December 2025. We anticipate that our study protocol will be feasible and that we will observe at least 80% participant retention at the follow-up assessments and their response to at least 75% of ecological momentary assessment signals. We hypothesize that depressive symptoms (distal factor) and negative affect (proximal factor) will be associated with increased alcohol use, and alcohol use will occur in lieu of disordered eating behaviors.

**Conclusions:**

Findings will help us understand distal and proximal factors leading to episodic alcohol use after undergoing MBS. This knowledge will allow us to construct better monitoring strategies for postoperative alcohol use within MBS programs and identify targets for intervention to reduce alcohol use after undergoing MBS.

**International Registered Report Identifier (IRRID):**

DERR1-10.2196/87209

## Introduction

Metabolic and bariatric surgery (MBS) is the most effective method for treating class 3 obesity, even compared to glucagon-like peptide-1 receptor agonist medications [1,2]. Despite the ability of MBS to result in significant weight loss and improvement in medical comorbidities, some patients experience adverse outcomes. One potential adverse outcome following MBS is the increased risk for alcohol use disorder (AUD). Up to 1 in 5 individuals may have an AUD 5 years after MBS, and most of these are considered new onset [3,4]. The increased risk of AUD appears to begin between 1 and 2 years after surgery and continues to rise as time elapses [3,5].

There are several possible explanations for why patients develop new problems with alcohol after surgery. The pharmacokinetics of alcohol change after MBS. For patients who have undergone sleeve gastrectomy (SG) or Roux-en-Y gastric bypass (RYGB; the most common procedure types), blood alcohol concentrations increase faster and peak higher [6-8]. Patients report increased sensitivity to the effects of alcohol after MBS [9-11]. MBS alters the ghrelin system, which may increase the rewarding effects of alcohol [12,13]. There is also a hypothesis that patients may be “transferring” their addiction [14,15]. Certain types of food act similarly in the brain as other addictive substances [16-23]. In functional magnetic resonance imaging studies, drug-related cues for those with a drug addiction and food-related cues for those with a “food addiction” show activation in the same areas of the brain (ie, the amygdala, orbitofrontal cortex, striatum, and insula) [16-23]. Patients identified as having preoperative problems with high-sugar or low-fat or high-glycemic-index foods (ie, foods thought to be addictive) were more likely to have a new onset of AUD after MBS [24]. However, other studies have not supported the addiction transfer hypothesis [25]. Previous work has also identified factors that are associated with increased risk of AUD following MBS. Characteristics associated with postoperative alcohol use and AUD include being male, younger age, preoperative alcohol use, other substance use, depression, and disordered eating behaviors [25,26].

Despite some useful scholarly work informing mechanisms of AUD, existing studies have several limitations. First, much of the work that has been done has examined preoperative factors while neglecting postoperative factors associated with alcohol use. Symptoms such as depression and disordered eating behaviors can change following MBS and may be worthwhile targets of intervention in the postoperative period [27,28]. Second, the preoperative factors that have been identified are broad and apply to a wide range of patients, making it challenging to identify high-risk individuals. Third, existing research has only evaluated average alcohol use with retrospective recall. Finally, no studies have examined proximal factors that lead to episodic alcohol use after MBS. Identifying postoperative factors associated with episodic alcohol use after MBS could lead to new targets for interventions to reduce alcohol use after MBS, thus preventing the development of postoperative AUD.

To identify proximal factors associated with episodic alcohol use (eg, mood and eating), we propose to use ecological momentary assessment (EMA). In EMA, participants respond to brief questions on their mobile device (eg, smartphone) in “real time” while involved in usual daily activities. EMA methodology improves the accuracy of reporting because it provides in-the-moment, real-world assessments, which capture nuanced patterns of alcohol use and the dynamics between use and proximal factors of episodic use that are unreliable with retrospective evaluation [29,30]. Measurement of proximal factors with distal factors (eg, psychiatric symptoms and drinking motives) could inform goals for various types of interventions to reduce alcohol use.

In this paper, we describe the study protocol of a project that seeks to characterize episodic alcohol use after MBS. The aim of this investigation is to determine the feasibility of the study protocol and understand the distal and proximal factors associated with postoperative episodic alcohol use.

## Methods

### Participants and Procedures

**Figure 1:**
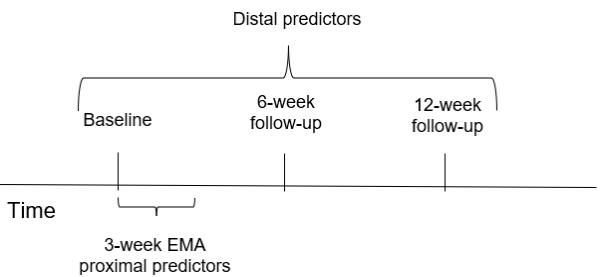
Timeline of participation. EMA: ecological momentary assessment.

The surgery types performed at this health system are RYGB and SG. We plan to enroll 100 participants who are between 6 months and 5 years after MBS. This will allow us to capture a range of drinking behavior, from reinitiation (to minimize recall bias) to continued use, by enrolling those who may have begun to escalate their frequency and amount of alcohol use [4,31]. Patients who underwent MBS at our health system will be randomly selected in batches and contacted via email with a brief description of the study and a link to a brief online eligibility survey through REDCap (Research Electronic Data Capture; Vanderbilt University) software, a secure and Health Insurance Portability and Accountability Act (HIPAA)–compliant web-based program. Patients will be asked if they have consumed alcohol since surgery. If so, they will report the frequency of use over the previous month (ie, not at all, once per month, 2-3 times per month, once per week, 2-3 times per week, 4-6 times per week, or every day). Patients will be eligible if they have consumed alcohol at least 2 to 3 times in the previous month, ensuring enrollment of those with current alcohol use. This criterion was also selected, given our goal to capture at least 2 drinking episodes during the EMA period. Once eligibility is determined, a research assistant will contact eligible patients via email or phone to schedule a time to complete informed consent and the baseline assessment. After obtaining participant consent to participate in the study, the research assistant will email a link to the baseline assessment, which will be completed through REDCap. Participants will also complete the assessment battery through REDCap at 6- and 12-week follow-ups (Figure 1). Participants will receive up to 5 reminders via email or phone if they have not completed each assessment. In addition, after completing the baseline assessment, each participant will complete 3 weeks of EMA. The EMA will include brief assessments (eg, 2-3 min) delivered twice per day to each participant’s smartphone. We propose to collect data each morning (eg, 8 AM) and in the evening before bedtime (eg, 8 PM); administering 2 surveys will reduce recall bias while also being cognizant of participant burden. Morning and evening assessments are frequently used in other EMA studies that have used a time-based approach [30]. An automatic push notification from the EMA app will be sent to the participant to signal when the items can be completed. Participants will be informed that the survey will be available on their dashboard 2 hours before the push notification is scheduled to allow for flexibility in the event that the participant wishes to complete it before receiving the push notification. Automatic reminders will be sent at 1 and 2 hours after the push notification if the survey has not yet been completed. Each signal will expire 4 hours after the push notification is sent. During the consent appointment, the research assistant will instruct participants on how to download and use the EMA app. If a participant misses multiple EMA signals in a row, the research assistant will attempt to contact the participant via email or phone.

### Measures: Distal Factors

Measures for the distal factors will be administered at baseline and at 6 and 12 weeks unless otherwise noted (Table 1).

**Table 1:** Measures at the baseline and 6- and 12-week assessments.

	Baseline	6 weeks	12 weeks
Demographics and background information	✓		
Preoperative substance use	✓		
Timing of postoperative substance use	✓		
Alcohol use (past 6 weeks)	✓	✓	✓
Hazardous alcohol use	✓		
Motives for alcohol use	✓	✓	✓
Cannabis use (past 6 weeks )	✓	✓	✓
Hazardous cannabis use	✓		
Depressive symptoms	✓	✓	✓
Anxiety symptoms	✓	✓	✓
Emotional eating	✓	✓	✓
Disordered eating behaviors	✓	✓	✓
Food addiction	✓		
Weight bias	✓		

#### Demographics and Background Information (Baseline Only)

Information collected at baseline will include age, sex and gender, race and ethnicity, height, weight, education level, current medications, lifetime history of psychiatric diagnoses, history of psychiatric treatment, and history of substance use treatment. We will also collect data from electronic health records, including surgery type (RYGB or SG) and preoperative height, weight, and BMI.

#### Preoperative Substance Use (Baseline Only)

Participants will report the frequency of alcohol use and types of alcohol consumed the year before MBS. They will report the frequency of cannabis use and the forms of cannabis used in the year before MBS. We will also collect data from the preoperative psychosocial evaluation regarding preoperative substance use (eg, type, frequency, and amount of substance use).

#### Timing of Postoperative Substance Use (Baseline Only)

Participants will report the number of months after MBS when they first consumed alcohol. Those reporting cannabis use after MBS will report the number of months since MBS when they first used cannabis.

#### Alcohol Use

Participants will respond to questions regarding their alcohol use over the previous 6 weeks. They will report types of alcohol consumed, frequency of alcohol use, typical number of drinks consumed per occasion, number of times they felt drunk, and number of binge drinking episodes (ie, 5 or more drinks for male individuals or 4 or more drinks for female individuals in 1 occasion).

#### Hazardous Alcohol Use (Baseline Only)

Participants will complete the Alcohol Use Disorders Identification Test [32]. The Alcohol Use Disorders Identification Test consists of 10 items that query the frequency and amount of alcohol use over the previous 6 months as well as the frequency of consequences from alcohol use. A score of 8 or greater suggests the presence of hazardous alcohol use.

#### Motives for Alcohol Use

The Drinking Motives Questionnaire-Revised [33] assesses respondents’ reasons for drinking across 4 categories over the past 6 weeks: conformity, coping, enhancement, and social. Each subscale consists of 5 items with responses ranging from 1 (strongly disagree) to 4 (strongly agree). Items from each subscale are added together for a total subscale score.

#### Cannabis Use

Although our primary outcome is alcohol use, we will also explore factors associated with cannabis use. Participants will respond to questions regarding their cannabis use over the previous 6 weeks. They will report frequency of cannabis use, forms of cannabis used, methods of ingestion, and average number of hours high or under the influence.

#### Hazardous Cannabis Use (Baseline Only)

The Cannabis Use Disorders Identification Test measures frequency of cannabis use over the past 6 months as well as consequences from cannabis use. A score of 8 or greater suggests the presence of hazardous cannabis use.

#### Depressive Symptoms

We will measure current (eg, past 2 weeks) depressive symptoms with the Patient Health Questionnaire-8 depression scale [34]. Participants will report the frequency of depressive symptoms from “not at all” to “every day.” Scores range from 0 to 24, with greater scores indicating greater severity. A score of 10 or greater indicates a high likelihood of current depression.

#### Anxiety Symptoms

We will also assess current (ie, past 2 weeks) anxiety symptoms with the Generalized Anxiety Disorder-7 [35]. Participants will report the frequency of anxiety symptoms from “not at all” to “every day.” Scores range from 0 to 21, with greater scores indicating greater severity. A score of 10 or greater indicates a high likelihood of current anxiety.

#### Emotional Eating

The Emotional Eating Scale will assess the urge to eat in response to 25 emotions that fall into 3 factors (ie, anger or frustration, anxiety, and depression) and can also be combined for a total overall score [36]. Greater scores indicate higher levels of emotional eating. This measure was validated among individuals with obesity [36] and has been previously used with individuals planning to undergo MBS [26,37].

#### Disordered Eating

Items from the Eating Disorder Examination-Questionnaire will be used to assess the frequency of disordered eating behaviors over the past 6 weeks. Participants will report whether they had a disordered eating behavior in the past 6 weeks (eg, loss of control, large portions, binge eating, grazing, and night eating) and, if so, the number of episodes each behavior occurred.

#### Food Addiction (Baseline Only)

Participants will complete the Yale Food Addiction Scale 2.0, which is a 35-item measure that assesses symptoms of food addiction that map onto the *Diagnostic and Statistical Manual of Mental Disorders, Fifth Edition* criteria for substance use disorders [38]. This measure has been validated for use with patients planning to undergo MBS [39].

#### Weight Bias

Weight bias will be assessed to collect preliminary data on postoperative weight bias to support future work. The Modified Weight Bias Internalization Scale is a 10-item measure in which participants respond to the degree to which they agree with statements regarding weight bias [40]. Higher scores indicate greater internalized weight bias.

### Measures: Proximal Factors

Proximal factors are measured during the EMA period. Table 2 presents the measures to be used in the daily EMA surveys. Completion of the complete set of items is expected to take less than 3 minutes.

**Table 2:** Measures for the ecological momentary assessment.

Construct		Morning measure		Evening measure
**Current mood**				
	Positive and Negative Affect Schedule–Short Form	10 items (Likert scale)	10 items (Likert scale)	
**Disordered eating**				
	Binge eating	Yes or no^a^	Yes or no^a^	
	Large portions	Yes or no^a^	Yes or no^a^	
	Loss of control	Yes or no^a^	Yes or no^a^	
	Grazing	Yes or no^a^	Yes or no^a^	
	Poor food choices	Yes or no^a^	Yes or no^a^	
	Emotional eating	Yes or no^a^	Yes or no^a^	
Alcohol use		Intention to use (yes, no, or maybe)		Actual use (yes or no)^b^
Alcohol use		Willingness to use (Likert scale)		—^c^
Cannabis use		Use the previous day (yes or no)^d^		—^c^

^a^If yes, what time did this begin?

^b^If yes, additional questions will query the type used, the amount, the time of use, and perceived intoxication.

^c^These items were not used on the evening survey.

^d^If yes, additional questions will query the form used, the time of use, and perceived high.

#### Mood

Participants will be asked to rate their current mood using the Positive and Negative Affect Scale–Short Form [41]. This measure has 5 items on each subscale (ie, positive and negative affect), allowing a range of affect to be assessed while remaining brief, which is important in EMA studies.

#### Disordered Eating

Participants will be asked if they engaged (yes or no) in 6 types of disordered eating behaviors (ie, binge eating, large portions, loss of control, grazing, poor food choices, and emotional eating) [42-44]. In the morning, the participant will report eating behaviors from the previous day. At the evening assessment, the participant will report on eating behaviors that occurred on that day. For each eating behavior endorsed, the participant will report the time at which the eating behavior began.

#### Alcohol Use

Participants will be presented with an image and description of a standard drink on every survey. At the morning assessment, participants will indicate whether they intend to use alcohol that day (yes, no, or maybe). They will also be asked about their willingness to drink (eg, “If somebody offered you an alcoholic beverage later today, would you drink it?”), to which they will respond using a scale from “definitely not” to “definitely yes.” They will also report the amount of alcohol use on the previous day (morning survey) or on that day (evening survey). If alcohol consumption is reported, they will report the time they started their first drink and the time they finished their last drink (with the option to select that they are still drinking). Participants will also report the types of alcohol consumed and their level of intoxication (“not at all” to “very intoxicated”). These items have been used in previous EMA studies on mood and substance use [45,46].

#### Cannabis Use

On the morning survey, participants will respond to whether they used cannabis the previous day (yes or no). If yes, they will report on the form of cannabis used, the time of use, and how high they felt (on a scale from “not at all” to “very high”).

#### Power Analysis

Power estimates assume an α of .05, with 2-tailed tests. For the regression models, G*Power calculations indicate that with 100 participants, power to detect a moderate effect (Cohen f^2^=0.15) is 0.94; power to detect a small-to-moderate effect (Cohen f^2^=0.10) is 0.80 [47]. For the multilevel modeling, a rule of thumb for estimating models with random effects is approximately 50 higher-level units (participants) with 20 observations per unit (time points) [48]. Our sample size and sampling design exceed this recommendation. For survival analysis, we computed power to detect a difference in hazard for 2 groups (eg, time to first drink as a function of men and women), assuming exponentially distributed survival times and all individuals followed for the same duration [49]. As we cover the full interval during which individuals initiate alcohol use, we can assume a follow-up period of twice the average median lifetime (2.0). For a large effect size (*R*=2.00, the ratio of the median interval to event in group 1 vs group 2), 88 participants are required to attain power of 0.80; for a moderate effect size (*R*=1.75), 134 participants are needed. Notably, the primary goal of this project is to establish the feasibility and acceptability of the study methodology; therefore, for all analyses, we will focus on the magnitude and direction of effects.

### Ethical Considerations

This study is approved by the Henry Ford Health Institutional Review Board, where recruitment will take place (17061). Institutional review board approval was obtained in March 2024. To protect privacy, participants will be asked whether they consent to share their data in a repository. Participants who do not consent to this will not have their data shared. No identifying information of any participant will be shared or published. Participants will be compensated for their participation. They will receive US $50 for completion of each of the assessments at the baseline, 6-, and 12-week follow-ups. During the EMA, they will receive US $2 for each signal completed (up to US $4 per day) for a total of up to US $84 for the 21 days. Participants who complete at least 80% of the EMA signals will also receive a US $50 bonus.

## Results

### Overview

This study received funding from the National Institute on Alcohol Abuse and Alcoholism (R21 AA029423) in May 2023. The primary goal of this study is to evaluate the feasibility of the study design. We will calculate the enrollment rate (the number of those who complete the baseline assessment of those eligible) and retention rates at the 6- and 12-week follow-ups. We anticipate that at least 50% of the eligible individuals will enroll and that at least 80% will complete the 6- and 12-week follow-ups. We will also calculate adherence to the morning and evening assessments during the EMA period. We anticipate that 75% of the participants or more will have at least a 75% response rate to the EMA assessments across the full 3-week EMA period.

The second goal of this study is to determine distal and proximal predictors of alcohol use. We plan to measure alcohol use in several ways. Our primary outcome is alcohol use on a given day during the EMA period (yes or no), and the secondary outcomes include the number of drinks and hazardous alcohol use. We will also explore whether there are differences in predictors for planned versus unplanned drinking. Data collection began in November 2024 and will conclude in December 2025.

### Distal Predictors

We will conduct multiple regression analyses predicting alcohol outcomes at 6 and 12 weeks. Predictors will include baseline measures of depression, anxiety, emotional eating scores, number of food addiction symptoms, frequency of disordered eating behaviors (binge eating, loss of control eating, large portions, grazing, and unhealthy food choices), and drinking motives; we will control for sex and examine sex differences by testing interactions with sex. We will control for time since drinking reinitiation, given that there will be variability in timing. We hypothesize that negative emotionality will be associated with increased drinking in lieu of disordered eating. We will also produce path models that include data from all 3 time points (baseline and 6 and 12 weeks) in a single model within Mplus (Muthén & Muthén).

We will use continuous time survival analysis [49-52] to investigate initiation of alcohol use following MBS (time 0). We can predict time to first drink from time-invariant variables, such as sex and history of preoperative alcohol use.

### Proximal Predictors

To examine proximal predictors of drinking, we will use multilevel modeling [53,54], which accounts for clustering (clustering for days within each person) and permits varying numbers of observations to account for missing reports. Level 1 (L1) effects correspond to within-person effects (eg, proximal behaviors at the level of the day), and level 2 effects indicate between-person effects (eg, distal predictors such as sex or L1 variables such as eating behaviors or depression aggregated over time). Consistent with recommendations, level 2 effects will be grand mean centered, and L1 effects will be person mean centered, such that the L1 effect reflects a deviation from one’s own typical behavior [55,56]. Outcomes will include any drinking and amount of drinking as well as our measure of unplanned drinking; predictors will include same-day mood and eating behaviors. Moreover, we can use the survey data to code whether drinking precedes mood, follows mood, or occurs contemporaneously to examine directionality within a day; these categorical variables can be used in subsequent analyses. In addition, daily-level data can be lagged to examine whether there are mood effects on next-day alcohol use and vice versa. We will also examine daily associations among the mood and eating variables. We hypothesize that alcohol use will occur on days characterized by greater negative mood is present and in lieu of disordered eating, given that we expect maladaptive coping strategies (ie, alcohol use or disordered eating in response to negative mood) to be used at different times, and we can code directionality among mood and drinking and eating behaviors for use in analyses.

## Discussion

The primary goals of this study are to determine the feasibility of the study design and identify distal and proximal factors associated with episodic alcohol use after MBS. We believe our study design will be feasible and that we will meet our benchmarks for enrollment and retention in the waves as well as adherence to the EMA. Our benchmarks were established based on previous EMA work [57]. These findings will support future use of this EMA design among those who underwent MBS.

Our study will examine distal and proximal factors associated with alcohol use. We hypothesize that depressive symptoms (distal factor) and negative affect (proximal factor) will be associated with higher levels of drinking. As many as 50% of the individuals planning to undergo MBS report a history of depression [58-60]. Our team has found that although depressive symptoms improve in the first year after MBS, depressive symptoms return 1 to 2 years following MBS [27]. This is the same time frame in which we found that postoperative alcohol use is commonly initiated [31]. We will also explore whether alcohol consumption occurs in lieu of disordered eating behaviors. Previous qualitative work has suggested that patients who underwent MBS do not consume alcohol and food together [61]. This study will extend this through a more thorough quantitative investigation. We will also determine whether patients consume alcohol in response to depression and negative affect in lieu of disordered eating behaviors. In addition, we plan to examine whether those who consume alcohol in lieu of disordered eating are more likely to engage in hazardous drinking. If affirmative, this would provide support for the addiction transfer hypothesis [25].

There are several strengths of this study, including that it will be the first to provide a granular examination of episodic alcohol use among individuals who underwent MBS. We are capturing real-world data at the time events are occurring, which improves the rigor of previous work that has relied on retrospective recall. The EMA design also allows for the temporal examination of mood, eating, and alcohol use, which can test the addiction transfer hypothesis in a way that has not previously been done. In addition, it is novel to look at intention to drink (ie, planned vs unplanned drinking and willingness to drink). However, it is important to note several limitations. First, because we are capturing real-world behavior, it is possible that there may be a low number of drinking episodes that occur during the EMA period. To prevent this, our recruitment will be targeted to those who endorse more frequent drinking behaviors (eg, at least 2-3 times per month). In addition, affect can fluctuate throughout the day. Because of the study design, we may not capture affect immediately preceding drinking. However, this work will support future investigation of these variables in a study with greater resources, allowing a more rigorous design among a larger sample.

This line of research will help us understand distal and proximal factors leading to episodic alcohol use after MBS. The results from this project will inform a future proposal with longer-term follow-up that will examine the progression from reinitiation of alcohol use to heavy use to the development of an AUD. This knowledge will allow us to construct better monitoring strategies for postoperative alcohol use within MBS programs. Findings will also assist in the development of interventions to reduce negative affect and alcohol use after MBS. By reducing postoperative alcohol use, we can prevent AUDs in this vulnerable, high-risk population.

## Appendix

Multimedia Appendix 1 Peer review report from AA-2 - Epidemiology, Prevention and Behavior Research Study Section, National Institute on Alcohol Abuse and Alcoholism Initial Review Group (National Institutes of Health, USA).

## Data Availability

Data sharing is not applicable to this paper as no datasets were generated or analyzed during this study.

## Abbreviations

AUD: alcohol use disorder
EMA: ecological momentary assessment
HIPAA: Health Insurance Portability and Accountability Act
L1: level 1
MBS: metabolic and bariatric surgery
REDCap: Research Electronic Data Capture
RYGB: Roux-en-Y gastric bypass
SG: sleeve gastrectomy
