# Supplementary material for: Factors Associated With Alcohol Use After Metabolic and Bariatric Surgery: Protocol for an Ecological Momentary Assessment
Source: JMIR Res Protoc. 2026 Jan 14;15:e87209. doi: 10.2196/87209 (PMC12853087; doi:10.2196/87209)
Supplement: Multimedia Appendix 1 [file resprot_v15i1e87209_app1.pdf]

SUMMARY STATEMENT
(Privileged Communication)

PROGRAM CONTACT:
I-Jen Castle
3018274406
i-jen.castle@nih.gov

Release Date: **11/24/2021**
Revised Date:

Application Number: **1 R21 AA029423-01A1**
Formerly: **1R21AA029423-01**

Principal Investigator

MATERO, LISA

Applicant Organization: HENRY FORD HEALTH SYSTEM

Review Group: **AA-2**
Epidemiology, Prevention and Behavior Research Study Section

Meeting Date: **10/25/2021**
Council: **JAN 2022**
Requested Start: **04/01/2022**
RFA/PA: **PA20-195**
PCC: **AE I**

Project Title: **Identifying Factors Influencing Alcohol Use after Bariatric Surgery: An Ecological Momentary Assessment**
SRG Action: **Impact Score:29**
Next Steps: **Visit https://grants.nih.gov/grants/next_steps.htm**
Human Subjects: **30-Human subjects involved - Certified, no SRG concerns**
Animal Subjects: **10-No live vertebrate animals involved for competing appl.**
Gender: **1A-Both genders, scientifically acceptable**
Minority: **1A-Minorities and non-minorities, scientifically acceptable**
Age: **3A-No children included, scientifically acceptable**

Project Year	Direct Costs Requested	Estimated Total Cost
1	125,000	197,671
2	150,000	237,205
TOTAL	275,000	434,876

ADMINISTRATIVE BUDGET NOTE: The budget shown is the requested budget and has not been adjusted to reflect any recommendations made by reviewers. If an award is planned, the costs will be calculated by Institute grants management staff based on the recommendations outlined below in the COMMITTEE BUDGET RECOMMENDATIONS section.

1R21AA029423-01A1 Matero, Lisa

SCIENTIFIC REVIEW OFFICER'S NOTES

RESUME AND SUMMARY OF DISCUSSION: The PI of this resubmitted application proposes to identify the distal (from baseline) and proximal (from ecological momentary assessment, EMA) factors that contribute to re-initiation and episodic alcohol use following bariatric surgery. The application has retained its previous strength. One in 5 patients develop an alcohol use disorder (AUD) following bariatric surgery which makes proposed research highly significant. The team of investigators poses relevant expertise in areas, are highly productive, and have experience in conducting research in the targeted population. The choice of sequential mixed methods is appropriate for the exploratory aims. The resubmission was somewhat responsive to the previously voiced issues. More details are given on the expertise and roles of the research team members. The recruitment period for the study is now 3 years instead of 6-months, clarifications were added to the data analytical plan. The remaining concerns included somewhat limited innovative aspect of the project especially study methodology. Lack of details on how this project contributes above and beyond of already funded R34. Additional concerns included lack of analysis to uncouple mood driven eating and alcohol use. Not all the reviewers agreed about the potential public health impact of the project as while alcohol misuse is a concern in these patients, the proportion of this patients in overall population is small. The above-mentioned concerns deterred interest with the proposal for some members of the review committee. The proposal was placed between Outstanding and Very Good range**.**

DESCRIPTION (provided by applicant):

Bariatric surgery is the most effective weight loss treatment for patients who are severely obese; however, 1 in 5 patients develop an alcohol use disorder (AUD) after undergoing surgery. After surgery, changes in metabolism, hormone levels, and behaviors alter the rewarding effects of alcohol while concurrently changing its absorption rate, putting patients at significantly elevated risk of hazardous drinking. To better understand the development of AUDs after bariatric surgery, we must first identify factors leading to re-initiation of alcohol use and escalation to heavy drinking. Promising areas include mood and eating behaviors. The purpose of the proposed study is to identify the distal and proximal factors that contribute to re-initiation and episodic alcohol use following bariatric surgery. We will use an exploratory sequential mixed methods approach and first obtain qualitative data to inform the quantitative phase. In the qualitative phase, we will conduct interviews of patients who are between 6 months and 3-years post-bariatric surgery (N= 30) to identify the reasons they consumed alcohol after bariatric surgery. We will also inquire about the frequency, amount, and the antecedents and consequences of episodic alcohol use (i.e., mood, eating behaviors). This information will be used to inform the quantitative data phase (i.e., finalize the constructs to assess, how and when to measure these variables, and the frequency of assessment). In the quantitative phase, patients (N= 100) will also be recruited between 6 months and 3-years post-surgery. Participants will complete measures of substance use, mood, and eating behaviors at baseline and at 6- and 12-weeks post-baseline to capture longer-term data to identify distal factors associated with alcohol use. Within the 12-week study period, participants will also be randomly assigned a 3-week period in which they will complete an ecological momentary assessment (EMA) design. The EMA design consists of brief, daily morning and evening assessments in “real-time” regarding emotions and behaviors (i.e., substance use, affect, and eating behaviors), which will allow us to identify the factors contributing to episodic alcohol use (i.e., proximal factors). We will also examine intended and unintended drinking. Our long-term goal is to better understand the progression from re-initiation of alcohol use, to heavy use, to development of an AUD, which will assist in targeting interventions to prevent AUDs. In addition to being the first study to examine distal and proximal factors of post-surgical alcohol use, this project contains multiple innovative components including an EMA design which allows for the examination of unintended

drinking. Further, given participants will have varying histories of alcohol use prior to surgery, we will explore whether distal and proximal predictors are differentially predictive of drinking among those with and without a history of heavy drinking prior to surgery. The proposed line of research is significant and relevant to NIH's mission because it will lead to tailored, effective interventions to reduce alcohol use and prevent the development of an alcohol use disorder among patients at high risk (i.e., those who undergo bariatric surgery).

PUBLIC HEALTH RELEVANCE

Bariatric surgery is the most effective treatment for severe obesity, yet more than 20% of individuals experience an alcohol use disorder after surgery due to metabolic and behavioral changes. Despite significant risks of problematic alcohol use among a growing number of individuals undergoing bariatric surgery, there is a lack of knowledge regarding the factors associated with post-surgical alcohol use. The proposed study moves the field forward and impacts public health by identifying the distal and proximal factors that contribute to re- initiation of and episodic alcohol use following bariatric surgery, which could lead to effective interventions to reduce post-surgical alcohol use and prevent the development of an alcohol use disorder.

CRITIQUE 1

Significance: 4
Investigator(s): 3
Innovation: 4
Approach: 4
Environment: 1

Overall Impact

The focus of this revised R21 proposal is to examine alcohol use onset and recurrence following bariatric surgery. Bariatric surgery patients are at very high risk for developing AUD. The aims of the current study are to explore why patients use alcohol following surgery (qualitative interviews), evaluate the feasibility of an EMA (twice daily) data collection design, and identify predictors of post-surgery drinking that are distal (from baseline) and proximal (from EMA, i.e., same-day mood and eating behaviors). The proposal identifies a significant issue, at least for the growing subset of the population undergoing bariatric surgery. However, the innovation of this exploratory R21 is somewhat diminished by the fact that the investigators are currently developing an intervention for the same population (through a current R34). Additional weaknesses include that the design is to recruit patients up to 3 years after surgery, and the ability to examine real-time covariation in mood, drinking, and eating is limited by twice daily surveys that will be aggregated to the day level.

1. Significance

Strengths

- Metabolic and behavioral changes following bariatric surgery are believed to contribute to 20% of patients developing an alcohol use disorder post-surgery
- For up to 70% of the patients with AUD, it is a new onset of AUD
- Bariatric surgery is becoming more common

Weaknesses

- Although AUD is high among bariatric surgery patients, is it not clear that a large percentage of people with AUD in the US are such patients.

2. Investigator(s)

Strengths

- PI Dr. Lisa Matero (Henry Ford Health System) has experience developing brief psychological interventions for patients undergoing bariatric surgery, and has conducted research on alcohol use after bariatric surgery
- Co-I Dr. Jordan Braciszewski (Henry Ford Health System) has expertise in technology-based substance use interventions and will lead qualitative analyses for Aim 1.
- Co-I Dr. Kristina Jackson (Brown University) has expertise in EMA and will serve as the statistician for the quantitative analysis for Aims 2 and 3.
- Consultant Dr. Roland Moore (PIRE) is a qualitative health researcher

Weaknesses

- Dr. Jackson's role on the project includes conducting all descriptive, multilevel, and survival analysis, although her level of effort is 15% for Year 2 (and 5% in Year 1). Path models are also described for aim 3. This is a large volume of work. There is no other data management or analysis support provided in the budget.

3. Innovation

Strengths

- Daily data to examine how mood and alcohol use co-vary among individuals who have had bariatric surgery
- EMA design to examine temporal ordering of mood and drinking
- Can examine when unintended eating and drinking occur

Weaknesses

- The proposal states that this will be the first study to examine the factors leading to post-surgical alcohol use. However, this seems like a stretch given that the investigators have an R34 study now to develop an intervention for this same issue. This weakens the innovation.
- The PI and Co-I Braciszewski's current R34 intervention mechanisms are not described. There is a comment that the R34 intervention does not currently target mood or eating behaviors, and that this R21 could inform the R34. But if the intervention does not target mood, is there a better candidate for why drinking is re-initiated?

4. Approach

Strengths

- Sequential mixed methods: qualitative data (N=30) about alcohol consumption after bariatric surgery, followed by quantitative phase (N=100) patients recruited 6 months to 3 years after surgery
- During the 12-week study period, randomly assigned to 3-week EMA
- Sex differences and interactions will be considered

Weaknesses

- Study recruitment will be 6 months to 3 years after surgery, despite the fact that most (3/4) begin drinking by 18 months after surgery.
- Twice daily EMA surveys will not capture the range of mood within a day, although this is a reasonable choice for an R21.
- The researchers plan to collect data multiple times per day but aggregate it to the daily level because of the sample size (according to the Introduction for Resubmission section). The power section does not explain this decision.
- The power analysis describes Group 1 vs. Group 2, which is not referenced elsewhere.
- Age is not described or considered, nor is current weight or BMI which may be especially important given the population

5. Environment

Strengths

- Henry Ford Health System has adequate resources, including approximately 700 bariatric surgery patients per year

Weaknesses

- Non noted.

Study Timeline

Strengths

- Not noted.

Weaknesses

- None noted.

Protections for Human Subjects

Acceptable Risks and/or Adequate Protections

- Plan in place

Data and Safety Monitoring Plan (Applicable for Clinical Trials Only):

Acceptable

- o DSMP included

Inclusion Plans

- Sex/Gender: Distribution justified scientifically
- Race/Ethnicity: Distribution justified scientifically
- For NIH-Defined Phase III trials, Plans for valid design and analysis:
- Inclusion/Exclusion Based on Age: Distribution justified scientifically
- ages 18 and older, population of individuals undergoing bariatric surgery (est. 80% women and 30% Black)

Resubmission

- The investigators clarified expertise of the team, including Co-I Braciszewski for qualitative analysis and Co-I Jackson for survival analysis. The recruitment period was changed to 6 months to 3 years post-surgery (instead of 1-3 years). Additional clarification about aggregating to the day-level for analysis is provided in the introduction, the PANAS was added as a measure of affect, and other substance use was removed.

Resource Sharing Plans

Acceptable

- Data sharing agreement proposed

Budget and Period of Support

Recommend as Requested

CRITIQUE 2

Significance: 2
Investigator(s): 2
Innovation: 3
Approach: 3
Environment: 1

Overall Impact

This is a resubmission of a R21 proposal to explore the factors that might contribute to problematic alcohol use and transition to alcohol use disorder (AUD) following bariatric surgery. Bariatric surgery patients are at higher-than-average risk for AUD prior to surgery, and this risk more than double after surgery. As such they represent a population of interest. The investigators plan to use a sequential mixed methods design, including patient interviews and ecological momentary assessment (EMA) to inform future large-scale mechanistic research and/or intervention research in this population. Although the use of EMA is ideally suited for exploring possible relationships among key variables of eating, mood, and alcohol use, the protocol could be more impactful if augmented with additional variables (possibly informed by interviews) and design choices (e.g., paired random and event contingent designs). These would be easy to implement, and overall, the potential value outweighs any concerns raised here.

1. Significance

Strengths

- Bariatric surgery patients have high rates of AUD.
- Rates of AUC in this patient group increase following surgery, but unclear why.
- 70% of cases are new onset following surgery.

Weaknesses

- No major weaknesses noted.

2. Investigator(s)

Strengths

- PI Matero has extensive experience clinically and conducting research on behavioral health in primary medical settings, particularly with this patient population
- PI Matero has shifted emphasis from practice to research and is picking up steam, highly productive in recent years.
- Co-I Jackson has exceptional expertise with intensive longitudinal study of alcohol use
- Co-I Braciszewski and Consultant Moore bring needed expertise in qualitative analysis.

Weaknesses

- None noted.

3. Innovation

Strengths

- Application of these methods in this population is a good pairing.
- Examining relationship between unplanned eating episodes and drinking is likely to be informative.

Weaknesses

- Ambulatory assessment designs that gathered more data would likely be informative.
- Potential value in uncoupling mood and assessments of eating and alcohol use (e.g., event contingent (e.g., report after every episode of food consumption) if really want to understand directionality.
- Given qualitative expertise on team, this would be a good opportunity to collect qualitative data during EMA assessments.

4. Approach

Strengths

- Varying time of participation after surgery is good for exploratory study.
- Sequential Mixed methods are good choice for study's exploratory aims.

Weaknesses

- Power analyses are missing for multilevel models. This is less of a concern given the exploratory nature of this mechanism and project.
- Meta-analyses show that negative affect is weakly associated, if at all, with drinking on day and momentary report. Planned analyses should also focus on positive affect, craving, boredom and audition other possible variables to flesh out possible predictors for future R01 work. In other words, given the nature of the project exploring more possible pathways to drinking would be good. Ideally would use qualitative data to augment planned EMA battery.
- No rationale given why daily lagged associations would be expected.
- Timeline Follow-Back is reasonable for average quantity but has been shown to be poor for patterning of substance use, alcohol in particular. Not sure it is useful for a "detailed picture of alcohol use."

5. Environment

Strengths

- The Henry Ford Health System has a large population of eligible post-surgery patients (n=700 per year) that should be adequate for the study.
- Brown University's CAAS is well-resourced to support quantitative analyses of study.

Weaknesses

- None Noted.

Study Timeline

Strengths

- Timeline is acceptable.

Weaknesses

- Timing of EMA design follows shortly after qualitative interviews, making it less likely that they will influence additional EMA items.

Protections for Human Subjects

Acceptable Risks and/or Adequate Protections

- This is generally a low-risk study.

Data and Safety Monitoring Plan (Applicable for Clinical Trials Only):

Acceptable

- Detailed plan in place.

Inclusion Plans

- Sex/Gender: Distribution justified scientifically
- Race/Ethnicity: Distribution justified scientifically
- For NIH-Defined Phase III trials, Plans for valid design and analysis: Not applicable
- Inclusion/Exclusion Based on Age: Distribution justified scientifically
- Planned 50% African American/Black and Female

Resource Sharing Plans

Acceptable

- Although the application does not mention it explicitly, presumably the quantitively data (i.e., that can be deidentified) would be required to be shared via NIMH Data Archive (NDA).

Budget and Period of Support

Recommend as Requested

CRITIQUE 3

Significance: 3
Investigator(s): 1
Innovation: 3
Approach: 2
Environment: 1

Overall Impact

This is a resubmission of R21 application from an early-stage investigator that is trained as a clinical psychology and is holds an Assistant Scientist position at Henry Ford Health System. The purpose of the proposed study is to identify the distal and proximal factors that contribute to re-initiation and episodic alcohol use following bariatric surgery. The resubmission was responsive to reviewer's critiques. The application now includes figures that help clarify the proposed research design, timing of data collection is addressed, additional measures to assess mood are included, examination of other substance use has been removed, and more detailed data analytic plan has been included. Overall, the proposed study is of public health significance given its potential to identify distal and proximal factors that contribute to problematic alcohol use following bariatric surgery. Study findings have the potential to elucidate factors influencing re-initiation and episodic alcohol use in a population at high risk for AUDs and could lead to a new just-in-time adaptive intervention targeting drinking among bariatric patients. The investigative team is strong with complimentary expertise relevant to all aspects of the proposed study and experience in conducting studies with the target population. The study utilizes psychometrically sound and widely used self-report measures to assess the constructs of interest. The data analytic plan is well-reasoned and appropriate to accomplish the specific aims of the study. Minor weaknesses included a target population in which risk of problematic alcohol use is substantially high, yet bariatric patients remain a limited amount of the total U.S. population. While the study methods are not in and of themselves innovative, examining the distal and proximal factors that lead to re-initiation of and continued alcohol use following bariatric surgery, including unintended drinking is new. Overall, enthusiasm for this application is high.

1. Significance

Strengths

- Bariatric surgery is the most effective treatment for severe obesity, yet more than 20% of individuals experience an alcohol use disorder after surgery due to metabolic and behavioral changes.
- Despite significant risks of problematic alcohol use among a growing number of individuals undergoing bariatric surgery, knowledge regarding the factors associated with post-surgical alcohol use is scarce.
- The goal of the proposed study is to examine alcohol use and mood following bariatric surgery utilizing a mixed methods approach, specifically exploratory qualitative interviews, and EMA.
- The proposed study is of public health significance given its potential to identify distal and proximal factors that contribute to problematic alcohol use following bariatric surgery.
- Results from this study will help us understand factors influencing re-initiation and episodic alcohol use in a population at high risk for AUDs and could lead to a new just-in-time adaptive intervention targeting antecedents of episodic alcohol use rather than just re-initiation.
- Results from this R21 project will inform a future R01 proposal that will inform a future R01 project that will identify pathways to alcohol use following bariatric surgery.

Weaknesses

- While risk of problematic alcohol is a substantial problem in this growing population, it remains a limited amount of the general population.

2. Investigator(s)

Strengths

- The investigative team have complimentary expertise relevant to all aspects of the proposed study and experience in conducting studies with the target population.

- Pi Matero is an early-stage investigator that is trained as a clinical psychology and holds an Assistant Scientist position at Henry Ford Health System.

- PI has experience in bariatric surgery, eating behaviors, and substance use and has a record of publications in this area. Dr. Matero is also a Licensed, Board Certified Clinical Health Psychologist and has previously conducted pre-surgical psychological evaluations and brief interventions for patients undergoing bariatric surgery.

- Co-I Braciszewski has experience in qualitative methods and substance use and will lead the qualitative analyses while consultant Moore who has extensive expertise in qualitative methods will provide input on the qualitative aspects of the study.

- Co-I Jackson will provide expertise on EMA. She is also an expert in longitudinal and survival analyses and will serve as the statistician for the quantitative analyses.

- PI Matero and Co-I Braciszewski currently collaborate on an NIAAA funded R34 that is examining a technology-based intervention to reduce alcohol use after bariatric surgery.

- The investigative team has a history of previous collaborations.

Weaknesses

- None noted

3. Innovation

Strengths

- This will be the first study to examine the distal and proximal factors that lead to re-initiation of and continued alcohol use following bariatric surgery

- The examination of unintended drinking in this population via EMA is also novel.

Weaknesses

- While the study methods are not in and of themselves innovative, examining the distal and proximal factors that lead to re-initiation of and continued alcohol use following bariatric surgery, including unintended drinking, is new.

4. Approach

Strengths

- The proposed study will use an exploratory sequential mixed methods approach and first obtain qualitative data (n=30) to inform the quantitative phase (n=100).
- The contribution of EMA in advancing science via the study design is well articulated. EMA will allow investigators to identify the directionality of mood, substance use, and eating behaviors in this understudied population.
- The examination of unintended drinking is a strength.
- The study utilizes psychometrically sound and widely used self-report measures to assess the constructs of interest.
- The data analytic plan is well-reasoned and appropriate to accomplish the specific aims of the study.
- The application identifies how potential problems will be addressed, what alternative strategies may be implemented, or which are benchmarks for success.

Weaknesses

- Self-report measures of alcohol use will lend itself to social desirability, particularly in population where alcohol use is highly discouraged.

5. Environment

Strengths

- The scientific resources and intellectual environment at the Henry Ford Health System is an excellent resource to successfully accomplish the proposed study aims. The investigative team appears to have the necessary institutional support, equipment, and other physical resources to implement the proposed research activities effectively.
- Letter of support from Division Head of General Survey indicates full support from the Bariatric Surgery Program at Henry Ford Health System.

Weaknesses

- None noted

Study Timeline

Strengths

- Time appears appropriate to accomplish the study aims.

Weaknesses

- None noted

Protections for Human Subjects

Acceptable Risks and/or Adequate Protections

- Risk to human subject, adequacy of protection, potential benefits of the proposed research to participants and others, and importance of knowledge to be gained at all adequately addressed.

Data and Safety Monitoring Plan (Applicable for Clinical Trials Only):

Not Applicable (No Clinical Trials)

Inclusion Plans

- Sex/Gender: Distribution justified scientifically
- Race/Ethnicity: Distribution justified scientifically
- For NIH-Defined Phase III trials, Plans for valid design and analysis:
- Inclusion/Exclusion Based on Age: Distribution justified scientifically

- Sex/gender, race/ethnicity, and age are scientifically justified. Investigative teams addresses how they will make an effort to ensure a sample that is representative of the target population.

Resubmission

- Resubmission was responsive to reviewer's critiques.

Resource Sharing Plans

Acceptable

- The inclusion of plans submits and share data with NIAAA Data Archive should also be presented.

Budget and Period of Support

Recommend as Requested

THE FOLLOWING SECTIONS WERE PREPARED BY THE SCIENTIFIC REVIEW OFFICER TO SUMMARIZE THE OUTCOME OF DISCUSSIONS OF THE REVIEW COMMITTEE, OR REVIEWERS' WRITTEN CRITIQUES, ON THE FOLLOWING ISSUES:

PROTECTION OF HUMAN SUBJECTS: ACCEPTABLE

INCLUSION OF WOMEN PLAN: ACCEPTABLE

INCLUSION OF MINORITIES PLAN: ACCEPTABLE

INCLUSION ACROSS THE LIFESPAN: ACCEPTABLE

COMMITTEE BUDGET RECOMMENDATIONS: The budget was recommended as requested.

SCIENTIFIC REVIEW OFFICER'S NOTES:
The application does not include details on plans to submit human subjects related data to NIAAA data repository as outlined in NOT-AA-19-020.

Footnotes for 1 R21 AA029423-01A1; PI Name: Matero, Lisa

NIH has modified its policy regarding the receipt of resubmissions (amended applications).See Guide Notice NOT-OD-18-197 at https://grants.nih.gov/grants/guide/notice-files/NOT-OD-18-197.html. The impact/priority score is calculated after discussion of an application by averaging the overall scores (1-9) given by all voting reviewers on the committee and multiplying by 10. The criterion scores are submitted prior to the meeting by the individual reviewers assigned to an application, and are not discussed specifically at the review meeting or calculated into the overall impact score. Some applications also receive a percentile ranking. For details on the review process, see http://grants.nih.gov/grants/peer_review_process.htm#scoring.

MEETING ROSTER

Epidemiology, Prevention and Behavior Research Study Section
National Institute on Alcohol Abuse and Alcoholism Initial Review Group
NATIONAL INSTITUTE ON ALCOHOL ABUSE AND ALCOHOLISM
AA-2
10/25/2021 - 10/26/2021

Notice of NIH Policy to All Applicants: Meeting rosters are provided for information purposes only. Applicant investigators and institutional officials must not communicate directly with study section members about an application before or after the review. Failure to observe this policy will create a serious breach of integrity in the peer review process, and may lead to actions outlined in NOT-OD-14-073 at https://grants.nih.gov/grants/guide/notice-files/NOT-OD-14-073.html, NOT-OD-15-106 at https://grants.nih.gov/grants/guide/notice-files/NOT-OD-15-106.html, and NOT-OD-18-115 at https://grants.nih.gov/grants/guide/notice-files/NOT-OD-18-115.html, including removal of the application from immediate review.

CHAIRPERSON(S)

MCGUE, MATTHEW K., PHD
PROFESSOR
DEPARTMENT OF PSYCHOLOGY
INSTITUTE OF HUMAN GENETICS
UNIVERSITY OF MINNESOTA
MINNEAPOLIS, MN 55455

MEMBERS

CADIGAN, JENNIFER M, PHD *
SENIOR FELLOW
PSYCHIATRY AND BEHAVIORAL SCIENCES
UNIVERSITY OF WASHINGTON
SEATTLE, WA 98115-6671

CAETANO, RAUL, PHD, MD, MPH
SENIOR RESEARCH SCIENTIST
PREVENTION RESEARCH CENTER
PACIFIC INSTITUTE FOR RESEARCH AND EVALUATION
OAKLAND, CA 94612

CANO, MIGUEL ANGEL, MPH, PHD
ASSOCIATE PROFESSOR
DEPARTMENT OF EPIDEMIOLOGY
FLORIDA INTERNATIONAL UNIVERSITY
MIAMI, FL 33199

DER-AVAKIAN, ANDRE, PHD *
ASSISTANT PROFESSOR
DEPARTMENT OF PSYCHIATRY
UNIVERSITY OF CALIFORNIA, SAN DIEGO
SAN DIEGO, CA 92093

FINK, BRANDI C, PHD *
ASSOCIATE PROFESSOR
DEPARTMENT OF PSYCHIATRY
UNIVERSITY OF NEW MEXICO HEALTH SCIS CTR
ALBUQUERQUE 87131
MEXICO

FURR-HOLDEN, C. DEBRA M., PHD
PROFESSOR
DEPARTMENT OF EPIDEMIOLOGY AND BIOSTATISTICS
COLLEGE OF HUMAN MEDICINE
MICHIGAN STATE UNIVERSITY
FLINT, MI 48502

HANSON, JESSICA D, PHD *
ASSISTANT PROFESSOR
UNIVERSITY OF MINNESOTA DULUTH
DULUTH, MN 55812

KERR, DAVID C. R., PHD
ASSOCIATE PROFESSOR
DEPARTMENT OF PSYCHOLOGY
SCHOOL OF PSYCHOLOGICAL SCIENCE
OREGON STATE UNIVERSITY
CORVALLIS, OR 97331

KEYES, KATHERINE MARGARET, PHD
ASSOCIATE PROFESSOR
DEPARTMENT OF EPIDEMIOLOGY
MAILMAN SCHOOL OF PUBLIC HEALTH
COLUMBIA UNIVERSITY
NEW YORK, NY 10032

MILLER, MARY ELIZABETH, PHD *
ASSISTANT PROFESSOR
DEPARTMENT OF PSYCHIATRY
UNIVERSITY OF MISSOURI-COLUMBIA
COLUMBIA, MO 65202

PATRICK, MEGAN ELIZABETH, PHD
RESEARCH PROFESSOR
INSTITUTE FOR SOCIAL RESEARCH
UNIVERSITY OF MICHIGAN
ANN ARBOR, MI 48109

RILEY, EDWARD P, PHD *
PROFESSOR
CENTER FOR BEHAVIORAL TERATOLOGY
DEPARTMENT OF PSYCHOLOGY
SAN DIEGO STATE UNIVERSITY
SAN DIEGO, CA 92120

SANCHEZ, MARIANA, PHD
ASSISTANT PROFESSOR
DEPARTMENT OF HEALTH PROMOTION AND
DISEASE PREVENTION
FLORIDA INTERNATIONAL UNIVERSITY
MIAMI, FL 33199

SARTOR, CAROLYN E, PHD *
ASSOCIATE PROFESSOR
DEPARTMENT OF PSYCHIATRY
YALE SCHOOL OF MEDICINE
NEW HAVEN, CT 06511

STOCKMEIER, CRAIG ALLEN, PHD *
PROFESSOR
DIVISION OF NEUROBIOLOGY & BEHAVIORAL RESEARCH
UNIVERSITY OF MISSISSIPPI MEDICAL CENTER
JACKSON, MS 39216

SUBICA, ANDREW MAKOTO, PHD
ASSISTANT PROFESSOR
SOCIAL MEDICINE, POPULATION, AND PUBLIC HEALTH
CENTER FOR HEALTHY COMMUNITIES
UNIVERSITY OF CALIFORNIA, RIVERSIDE
RIVERSIDE, CA 92521

WRIGHT, AIDAN GREGORY CRAVER, PHD *
ASSOCIATE PROFESSOR
DEPARTMENT OF PSYCHOLOGY
UNIVERSITY OF PITTSBURGH AT PITTSBURGH
PITTSBURGH, PA 15218

SCIENTIFIC REVIEW OFFICER

GHAMBARYAN, ANNA, MD, PHD
SCIENTIFIC REVIEW OFFICER
EXTRAMURAL PROJECT REVIEW BRANCH
OFFICE OF EXTRAMURAL ACTIVITIES
NATIONAL INSTITUTE ON ALCOHOL ABUSE AND
ALCOHOLISM
NATIONAL INSTITUTES OF HEALTH
BETHESDA, MD 20892

EXTRAMURAL SUPPORT ASSISTANT

STRINGFIELD, DONNA
EXTRAMURAL SUPPORT ASSISTANT
OFFICE OF EXTRAMURAL ACTIVITIES
NATIONAL INSTITUTE ON ALCOHOL ABUSE AND
ALCOHOLISM
NATIONAL INSTITUTES OF HEALTH
6700B ROCKLEDGE DRIVE, ROOM 1460B, MSC 6902
BETHESDA, MD 20892

* Temporary Member. For grant applications, temporary members may participate in the entire meeting or may review only selected applications as needed.

Consultants are required to absent themselves from the room during the review of any application if their presence would constitute or appear to constitute a conflict of interest.
